# Hot-Probe Characterization of Transparent Conductive Thin Films

**DOI:** 10.3390/ma14051186

**Published:** 2021-03-03

**Authors:** Alexander Axelevitch

**Affiliations:** Engineering Faculty, Holon Institute of Technology (HIT), Holon 5810201, Israel; alex_a@hit.ac.il; Tel.: +972-544-718-122

**Keywords:** dynamic hot-probe measurements, indium-tin oxide, aluminum-zinc oxide, magnetron co-sputtering

## Abstract

Transparent conductive oxide (TCO) thin films represent a large class of wide-bandgap semiconductors applied in all fields of micro- and optoelectronics. The most widespread material applied for the creation of TCO coatings is indium-tin oxide (ITO). At the same time, there are plurality trends to change the high-cost ITO on other materials, for example, on the ZnO doped by different elements such as Al, Mn, and Sb. These films require mobile and low-cost evaluation methods. The dynamic hot-probe measurement system is one of such techniques that can supplement and sometimes replace existing heavy systems such as the Hall effect measurements or the Haynes–Shockley experiments. The theoretical basis and the method of analysis of the recorded dynamic hot-probe characteristics measured at different temperatures were presented in this work. This method makes it possible to extract the main parameters of thin films. Commercial thin ITO films and new transparent conducting ZnO:Al layers prepared by magnetron co-sputtering were studied by the proposed method. The measured parameters of commercial ITO films are in agreement with the presented and reference data. In addition, the parameters of ZnO:Al thin films such as the majority charge carriers type, concentration, and mobility were extracted from dynamic hot-probe characteristics. This method may be applied also to other wide-bandgap semiconductors.

## 1. Introduction

Transparent conductive thin films play a very important role in microelectronics and optoelectronics nowadays. Thin films such as ITO (indium-tin oxide) have become the standard coating used in all sides of the micro- and optoelectronics. The ITO thin films found applications in solar cells, flat panel displays, flat transparent heaters, electrochromic windows, etc. At the same time, there are many trends to substitute the high-cost indium by other materials. Transparent conductive zinc oxide (ZnO) films are attractive semiconductor coatings that may substitute ITO films in high-temperature applications (higher than 700 K) [1]. Due to the impurity concentration and the thin films structure, these films can have a resistivity from 10^10^ to 10^−4^ Ω·cm [2]. There are many methods for producing doped ZnO films: thermal evaporation using an electron beam [3], sputtering in a DC plasma [2] and in a RF plasma [4], sputtering from a mosaic target [5], and simultaneous sputtering from two different targets [6]. The method of simultaneous sputtering from two targets was used in this work. This method allows for a fairly accurate control of the alloy metal percentage by controlling the power of the spray sources and the distance between the sputtering sources and the substrate.

All thin-film semiconductor materials require the fine control of their characteristics for successful applications. Transparent conductive oxides are wide-bandgap semiconductors, usually of the degenerate type, that is, having the high concentration of majority carriers. Their properties significantly depend on their growth conditions. Thus, the indium oxide, In_2_O_3_, has a bandgap of ≈3.7 eV [7]. To obtain enough high conductivity while maintaining high transparency, this material is applied with alloyed tin (Sn) in relation 5–10%. Sn produces interstitial bonds with oxygen inside the In_2_O_3_ structure by replacing the In^3+^ atoms on the Sn^2+^ and Sn^4+^, which exist either as SnO or SnO_2_ states, respectively. However, a predominance of the higher valency in Sn acts as an n-type donor releasing electrons to the conducting band. Thus, in ITO, both interstitial tin and oxygen vacancies contribute to the high conductivity [8].

Zinc oxide, ZnO, is a wide-bandgap semiconductor of class A_2_B_6_ with a direct bandgap of width ≈3.4 eV [9]. To increase the conductivity of ZnO thin films, doping with various material, such as Al, Sn, and other, was used [2,10]. Al-doped ZnO (AZO) is the most widespread transparent conductive coating (TCO) based on the ZnO. Due to the wide bandgap, perfect ZnO crystals have low conductivity due to the limited (low) number of intrinsic charge carriers at room temperature (RT). Therefore, defects begin to play a decisive role in the conductivity of wide-bandgap semiconductors. Thus, two type of defects, substitutional Al^3+^, replacing Zn^2+^, acting as donors and oxygen vacancies define electrical properties of AZO thin films. Table 1 represents the basic average values of the electrical parameters of ITO and AZO [8,11,12].

Properties of TCO coatings are influenced significantly by the deposition method because their conductivity is defined by the density and distribution of defects. Basic characteristics of these coatings such as transmittance in the required range and resistivity are defined by the type of semiconductor, bandgap of material, majority charge carrier’s concentration, and the mobility and lifetime of the majority carriers. Usually, these parameters may be measured by such conventional methods as the Hall effect measurement and the Haynes–Shockley experiment, which provide the required data. However, these methods are complex and too expensive. For example, the Hall measurements require the samples with very accurate geometry. Equipment for the Haynes–Shockley experiment uses the specific short-time switching systems for minority charge carrier’s excitation. Thus, these methods are available in a limited number of laboratories equipped by these high-cost systems only. The main requirements for evaluation methods are simplicity, efficiency, and sufficient accuracy [13]. Therefore, a simpler and faster method for evaluation of the mentioned parameters is required. The low-cost dynamic hot-probe method [13,14,15] for the measurement, recording, and extraction of the basic parameters of semiconducting TCO thin films is presented in this work. To verify the proposed method, the commercial ITO coatings and competing home-made AZO thin films prepared using the magnetron co-sputtering technique were investigated using the dynamic hot-probe method.

## 2. Materials and Methods

### 2.1. Experimental Details

**Figure 1:**
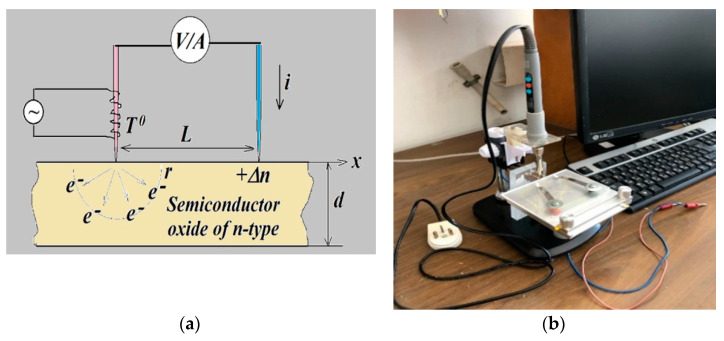
The laboratory setup for dynamic hot-probe measurements: (**a**) the principal operation scheme; (**b**) the laboratory home-made measurement setup.

Two types of transparent conductive coatings were investigated: the commercial ITO-coated glass slides with dimensions 25 × 75 mm^2^ and thickness of 1 mm of “Nanocs” and the glass slides of the same dimensions coated by thin ZnO films doped by Al with various concentrations. Sheet resistance and transmittance in the visible range of commercial ITO thin films are dependent on the film thickness. Usually, these films have a thickness of 15–300 nm, the sheet resistance of 5–100 Ω/sq, and the transmittance is over 85% [16]. The optical properties of the films were measured using the spectrophotometer UV-VIS “BioMate 3S”. The films’ topography was studied using the metallurgical microscope Hirox RH-2000. The surface resistivity was measured by the standard four-point method using the Macor-probe of MDC. Rough approximate estimation of the films’ thickness was carried out by weighing the substrates before and after deposition by using the analytical balance ASB-220-C2. In addition, the thickness of the films can be determined on the basis of the interference fringes of the measured transmittance characteristics. This approach was applied to estimate the thickness of a commercial ITO film. The dynamic hot-probe method was used for characterization of the type, concentration, and mobility of the major charge carriers in commercial ITO coatings and grown AZO thin films. Figure 1 represents the principal measurement scheme (a) and the laboratory home-made setup for dynamic hot-probe measurements (b). To provide measurements, we applied the Digital Multimeter 34405A of Agilent and the software “Keysight BenchVue” providing the real-time measurement and the recording of measured signals. The measurement duration was chosen from 30 to 90 s in order to avoid heating of the second (cold) electrode. It is known that the temperature of the cold electrode does not exceed room temperature by more than 20 °C during the specified time period when temperatures of the hot electrode do not exceed 300 °C [14].

The principle of operation of this technique is that the heated majority of charge carriers, electrons or holes, have a higher velocity of movement than the cold ones. As soon as we begin to heat one of two electrodes, joining the surface of the semiconductor, the major charge carriers run away from it. As a result, a potential difference arises between the hot and cold electrodes, the sign of which is determined by the type of major charge carriers. If the heated electrode is connected to the positive terminal of the measuring voltmeter, electronic conductivity will show a positive voltage, and a hole-type material will show a negative voltage. To short-circuit the hot and the cold electrodes with an ammeter, a current will begin to flow through it. The sign of the current is defined by the type of the major charge carriers. The bandgap of transparent conductive oxides is large; therefore, the growth in the concentration of intrinsic charge carriers due to the heating of the hot electrode will be vanishingly small. Therefore, only thermal running away of the heated charge carriers should be taken into account through the evaluation process. Analysis of the measured dynamic characteristics of voltage and current dependences on the temperature conditions allows us to determine the recombination rate, the concentration, and the mobility of majority carriers.

ZnO thin films alloyed with Al were deposited simultaneously by the magnetron co-sputtering using a VST service RF-DC sputtering system, model TESF-842, equipped with a turbomolecular pump enabling an ultimate pressure lower than 5·10^−7^ Torr [17]. The system includes three magnetrons of 2 inches in diameter and enables the simultaneous co-sputtering process using a 13.56 MHz, 300 W RF generator and two DC supplies of 1000 W each. All thin films were deposited at argon atmosphere with the pressure of 3–5 mTorr. Deposition duration was 30 min always. The RF power was supplied to the ZnO target, and the DC power was applied to the Al target. These thin films were grown at room temperature, and they were annealed after deposition in vacuum at 300, 400, and 500 °C through one hour. The amount of Al in the ZnO matrix was controlled by the relation of power applied to the Al target. We tried to grow the ZnO thin films alloyed with approximately 2% weight of aluminum. Evaluation of the thin films composition was provided using Energy Dispersive Spectrometry (EDS) system—Oxford Instruments X-Max^N^ detector (Czech Republic) completed with the electronic microscope FEG-SEM Tescan Mira 3.

### 2.2. Theoretical Background

Let us consider once more Figure 1a, the principal operation scheme of the hot-probe measurements. Charge carriers in semiconductors can be in two states: a state of static (thermodynamic) equilibrium and a non-equilibrium state, due to external influences that may be electric, magnetic, or temperature fields. The distribution of charged carriers on energy in the equilibrium state is defined by the Fermi–Dirac equation

(1) $$f_{0}\left(E\right)=\frac{1}{1+e^{\frac{E-E_{F}}{k_{B}T}}},$$

where *E_F_* is a Fermi energy, *T* is the absolute temperature in Kelvins, and *k_B_* is the Boltzmann constant. At the presence of external influences, the charge carriers system will be described by the non-equilibrium distribution function *f*(*E*) = *f*(*p,r,t*) dependent on energy (momentum, *p*), coordinates (*r*), and time (*t*). This function, *f*(*E*), takes into account all possible mechanisms by which the distribution function may be changed. In real materials, all charge carriers experience collisions with other particles, impurities, phonons, etc. So, the function *f*(*E*) should take into account the scattering of charged carriers due to collisions when the function comes to another equilibrium state. If the external influence is finished, the function will return to the initial state. By this way, the distribution function behavior may be presented by three different parts: an excitation, defined by the value of influencing external field; a steady state with slow current processes; and a relaxation part with a return to the initial state.

All these parts may be described by the same general equation taking into account all external and internal processes. In general, the behavior of charged carriers, in particular electrons, can be described by the well-known Boltzmann Transport Equation (BTE), which in differential form looks as follows [18,19]:

(2) $$\frac{\partial f}{\partial t}+\dot{k}\frac{\partial f}{\partial k}+\dot{r}\frac{\partial f}{\partial r}={\left(\frac{\partial f}{\partial t}\right)}_{Coll},$$

where the first summand on the left side represents the function variation in time, the second represents the influences of external fields, and the third shows the coordinate variation. The right part of the equation takes into account different types of collisions affecting the motion of particles: scattering by ionized impurities, scattering by neutral impurities, scattering by dislocations, and scattering by grain barriers, which is very important for polycrystalline semiconductor thin films [20]. The right side represents a scattering of the particles and, for simplicity, it may be presented using an electron relaxation time, *τ.* In the case when there are not external fields and charge carriers, return from the excited state to the initial state occurs as follows (relaxation time approximation):

(3) $${\left(\frac{\partial f}{\partial t}\right)}_{Coll}=-\frac{f-f_{0}}{\tau }.$$

This value, *τ*, depends on the dominant scattering mechanisms and may be found from experimentally measured dynamic hot-probe characteristics.

To evaluate the recorded dynamic hot-probe characteristics, it is necessary to transform the BTE into an equation without external electrical and magnetic fields; it must take into account only the temperature gradient affecting the movement of charge carriers. Only an internal electric field arises with a directed flow of charge carriers. We also assume that the material being measured is isotropic and the charge carriers flux occurs in only one *x* direction, where *x* denotes the *r* coordinate.

(4) $$F=m\frac{dv}{dt}=\hbar \frac{dk}{dt}=-qE_{ex},$$

where *E_ex_* is an external electrical field. According to Relation (4), the force related term in the full BTE (2) may be described as follows:

(5) $$\dot{k}\frac{\partial f}{\partial k}=-qE_{ex}v\frac{df}{dE_{ex}},$$

however, in the case of no external electrical field, this term will be vanished.

When the material is heated at a certain point on the surface, the charge carriers begin to move and, thus, they create a current and an electrical field in accordance with the following relationship [21]:

(6) $$E_{x}=\frac{1}{\sigma }j+\beta \frac{dT}{dx},$$

where *j* is the current density, σ is the conductivity of a material dependent on temperature, and β is the additional coefficient characterizing the thermo-electrical properties of the material. This coefficient represents the thermopower (the Seebeck coefficient) produced in the material under non-homogeneous heating due to charged carriers transport [22]. Evidently, this movement occurs only up to reaching of steady state when the current reaches a suitable value *j_s_* (saturation state) and our material will come to the new dynamic steady state (excited state). In this state, the created electrical field is defined by the thermal non-equilibrium and moving charge carriers:

(7) $$E_{x}=\frac{1}{\sigma }j_{s}+\beta \frac{dT}{dx}.$$

Equation (7) may be integrated on the distance *L*, which takes the follows result:

(8) $$U_{L}=\frac{L}{\sigma }j_{s}+\beta \Delta T.$$

Here, the conductivity of the sample may be described by the known formulae (the Drude equation):

(9) $$\sigma =\frac{1}{\rho }=q\mu n,$$

where μ is the mobility of charge carriers and *n* is its concentration. Both parameters, mobility and concentration of charge carriers, are functions of temperature. The mobility of charge carriers, μ, may be found using the well-known Einstein’s relation:

(10) $$D=\mu \frac{k_{B}T_{e}}{q},$$

where *D* is the diffusion coefficient of the major charge carriers, *k_B_* is the Boltzmann’s constant, and *T_e_* is an ambient temperature in K. Taking into account that the diffusion distance in our setup is equal to $L=\sqrt{D\tau }$, where τ is the measured relaxation time and substituting with Equation (10), we obtain:

(11) $$\mu =\frac{L^{2}q}{k_{B}T_{e}\tau }.$$

On the other hand, the mobility of charge carriers characterizes the scattering processes through the average scattering relaxation time, 〈τ〉:

(12) $$\mu ≅\frac{q\langle \tau \rangle }{m^{*}},$$

where m* represents an effective mass of electrons. All scattering processes occur simultaneously; therefore, the mobility may be presented by two summands representing the lattice, μ*_L_*, and impurity, μ*_i_*, scattering:

(13) $$\frac{1}{\mu }=\frac{1}{\mu_{L}}+\frac{1}{\mu_{i}}.$$

The expressions for these parameters may be presented in following form [23]:

(14) $$\mu_{L}\propto \frac{4q}{m^{\times }\sqrt{9\pi k_{B}}}T^{-1.5},$$

(15) $$\mu_{i}\propto \frac{8qk_{B}^{1.5}}{N_{i}m^{\times }\sqrt{\pi }}T^{1.5},$$

where *N_i_* is the concentration of ionized impurities. Mobility may be found from recorded hot-probe characteristics using a numerical differentiation method, for example, using the known approximate three-point formula of Lagrange [24]:

(16) $$\{\begin{array}{c} f^{\prime }\left(t_{0}\right)=\frac{1}{2\Delta t}\left[-3f\left(t_{0}\right)+4f\left(t_{0}+\Delta t\right)-f\left(t_{0}+2\Delta t\right)\right]\\ f^{\prime }\left(t_{0}+\Delta t\right)=\frac{1}{2\Delta t}\left[-f\left(t_{0}\right)+f\left(t_{0}+2\Delta t\right)\right]\\ f^{\prime }\left(t_{0}+2\Delta t\right)=\frac{1}{2\Delta t}\left[f\left(t_{0}\right)-4f\left(t_{0}+\Delta t\right)+3f\left(t_{0}+2\Delta t\right)\right] \end{array},$$

where a function *f* represents the measured voltage, *t_0_* is the first time-point of the decreasing function, and Δ*t* is the time-space between measured points. The thermopower may be found by the same way for suitable temperatures. 

Combining Equations (8) and (9), we obtain an equation describing the behavior of the studied material under hot-probe measurement conditions.

(17) $$U_{L}=\frac{L}{q\mu n}j_{s}+\beta \Delta T$$

When a heater comes in contact with the electrode, electrons run up into a semi-infinite space of a conductive matter. So, the current density will be related with the measured current, *i_s_*, according to the following equation:

(18) $$j_{s}=\frac{i_{s}}{2\pi r^{2}},$$

where *r* is a distance from the heated electrode (2π*r*^2^ being the surface area of the hemisphere). The propagation of electrons in the space shaped in the form of a thin disc is limited by the disc thickness. Therefore, Equation (18) transforms into the following approximated relation at the distance of integration:

(19) $$j_{s}=\frac{i_{s}}{2\pi dL}.$$

After substitution, Equation (17) transforms into the following final expression:

(20) $$U_{L}=\frac{i_{s}}{2\pi q\mu nd}+\beta \Delta T.$$

This equation, describing the system behavior in both excited and relaxed states, may be solved for the free charge carriers concentration:

(21) $$n=\frac{i_{s}}{2\pi q\mu d\left(U_{L}-\beta \Delta T\right)}.$$

Thus, one can calculate the concentration of charge carriers using the experimental data.

The concentration of free charge carriers depends significantly on the thickness of the studied thin film (see Equation (21)). Therefore, the method and accuracy of the thickness measurement will affect the results of our calculations. An error in determining the thickness leads to a deviation in the value of the concentration of charge carriers. Moreover, it is easy to show that the experimental error in determining the concentration of free charge carriers will always be less than the experimental error in measuring the thickness:

(22) $$\frac{\Delta n}{n}=-\frac{\Delta d}{\left(d+\Delta d\right)}.$$

Therefore, the thickness becomes a decisive factor in determining the accuracy of the thin film parameters extracted from the hot-probe characteristics.

A real experiment is a process driven by both variable controlled parameters and random variables that influence the expected results. In the case of dynamic hot-probe measurement, these casual random values can represent a small deviation of the temperature of the heated electrode from the specified one, a change in the contact of this electrode with the sample being measured, etc. Therefore, in order to reduce the influence of various random parameters on the shape of the recorded hot-probe characteristic, it is desirable to carry out several measurements under the same conditions. Recorded characteristics in tabular form should be averaged to use in the following calculations. Obviously, the measurement accuracy increases with the number of measurements. Usually, in order to achieve required process accuracy, it is sufficient to carry out three different measurements at the same temperature and process duration in different places of the studied thin film.

## 3. Results and Discussion

### 3.1. Commercial ITO Thin Films

**Figure 2:**
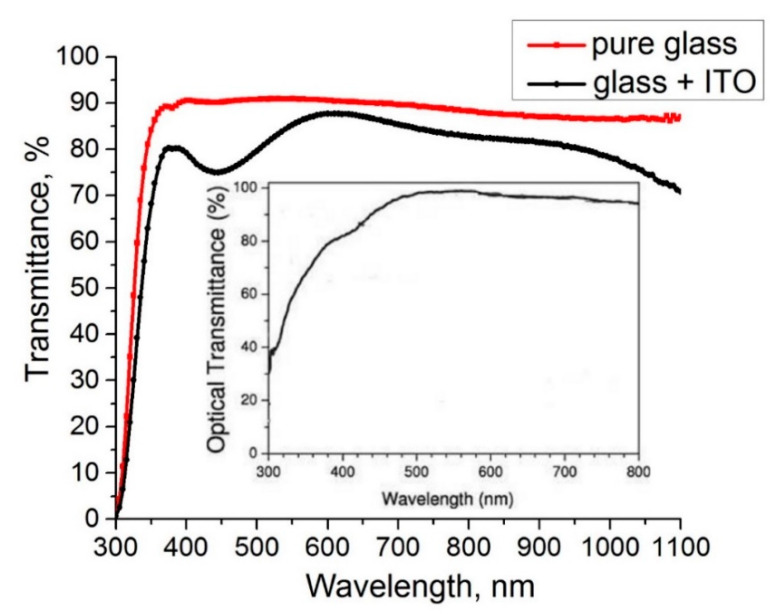
Measured transmittance characteristics of the commercial ITO on glass. The inset represents the absolute transmittance of ITO presented by the manufacturer [16].

First, we measured the optical properties and sheet resistance of glass slides coated by ITO because the accompanying documents obtained with the commercial slides present the average characteristics only. Figure 2 represents the measured transmittance characteristic of the ITO/glass system.

The inset shows the absolute transmittance characteristic of the ITO films produced by “Nanocs” [16]. The difference between the two curves in Figure 2 can be explained as follows: the manufacturer presents one of the measured or average transmittance characteristics, and the actual characteristics plotted on the graph always have some difference. The sheet resistance of the commercial ITO films measured using the four-point probe method was of *R_#_* = 8.3 Ω/sq, which correlates with the data of the manufacturer (10 Ω/sq).

To estimate the thickness of the commercial ITO film on the glass slide, the measured transmittance characteristics of this film (see Figure 2) should be considered. The measured spectrum demonstrates periodic oscillations of transmittance due to interference effects in the thin film. These effects are caused by multiple reflections of light from interfaces between the thin film, glass substrate, and air. The transmittance of this system may be described using the following equation [25]:

(23) $$T=\frac{\left[1-{\left(\frac{1-n}{1+n}\right)}^{2}\right]\left[1-{\left(\frac{n-n_{glass}}{n+n_{glass}}\right)}^{2}\right]}{1+{\left(\frac{1-n}{1+n}\right)}^{2}{\left(\frac{n-n_{glass}}{n+n_{glass}}\right)}^{2}-2\left(\frac{1-n}{1+n}\right)\left(\frac{n-n_{glass}}{n+n_{glass}}\right)cos\left(\frac{4\pi }{\lambda }nd\right)},$$

where *n_glass_* is the refractive index of the glass substrate, *n(*λ*)* is the refractive index of the film dependent on the light wavelength, λ, and *d* is the thickness. The maximum and minimum transmittance, according to Formula (23), are defined by the following relations: 2*nd* = mλ (m = 1, 2, 3…) and 2*nd* = (2m + 1)λ/2 (m = 0, 1, 2…) respectively. Then, one can choose the maximum or minimum points in the measured characteristics and calculate the thickness by choosing and changing the refraction coefficient. Verification should be made by calculation of the transmittance and comparing with the measured values. The calculated thickness of the ITO films was 150 ± 10 nm or ≈150 nm. Then, the resistivity of these films at room temperature can be estimated:

(24) $$\rho =R_{\#}\cdot d=0.83\cdot {1.5\cdot 10}^{-4}=1.25\cdot 10^{-4}\;\left[\Omega \cdot \mathrm{cm}\right].$$

**Figure 3:**
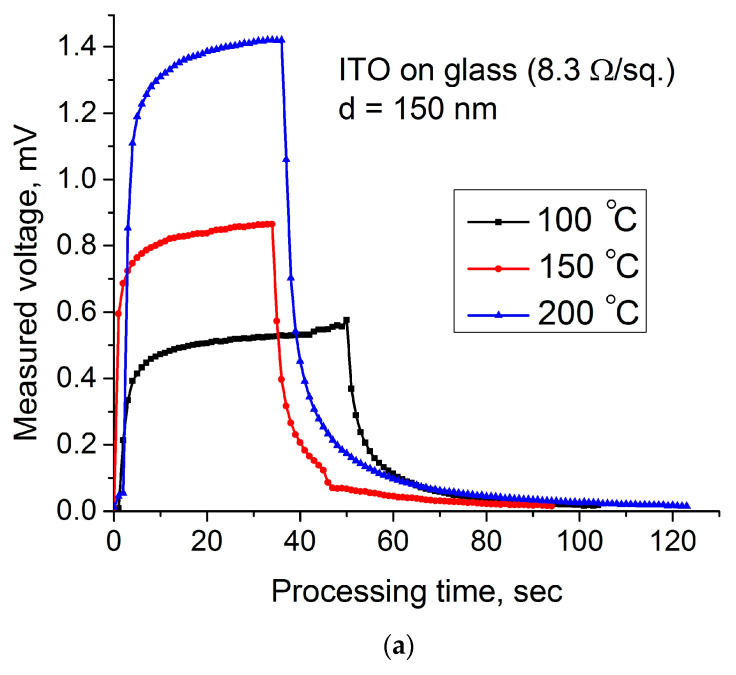

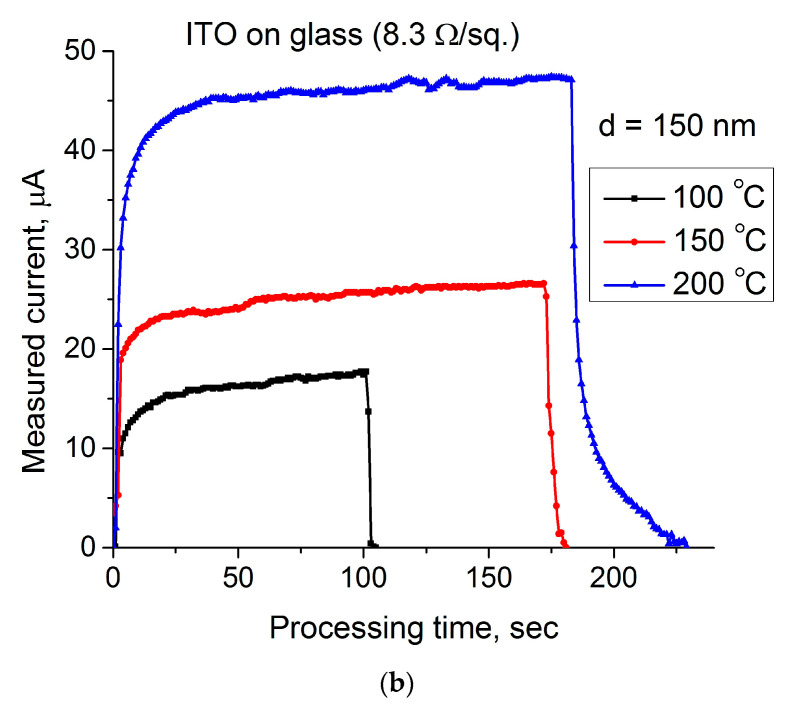
Dynamic hot-probe characteristics of the commercial ITO coatings: (**a**) a voltage measured between hot and cold electrodes; (**b**) a current between electrodes.

Figure 3 represents the dynamic hot-probe characteristics recorded on the commercial glass slides coated by the ITO.

Figure 3a illustrates the dependence of a hot-electrode voltage on the processing time for different temperatures, and Figure 3b shows the current measured between hot and cold electrodes, which were measured for the same temperatures. The curves shown in Figure 3a represent a positive voltage measured between hot and cold electrodes for different temperatures. This shows that the ITO coating is an n-type semiconductor. As shown, the dynamic curves repeat each other; therefore, all dynamic processes in the film are the same for different temperatures. In the recorded plots, three different areas can be identified: the region of a steep increase in voltage due to heating, the region of steady-state voltage, and the region of a sharp decrease in voltage when the heater is disconnected from the hot electrode. All these processes occur in the absence of some external electrical or magnetic fields, so they are driven by the temperature difference only. The steep rise of the voltage between electrodes and the current reflects a run up of hot electrons from the heated electrode. This rise happens very quickly due to the difference between rates of the heated and cold electrons. Evidently, these rates are proportional to the root square of the heating temperature. Therefore, a higher charge difference will be obtained for higher temperature, which is confirmed by measurements. After measurements at elevated temperatures and after the relaxation time, as shown in Figure 3, the properties of films return to their original state.

### 3.2. AZO Thin Films Prepared by Co-Sputtering

**Figure 4:**
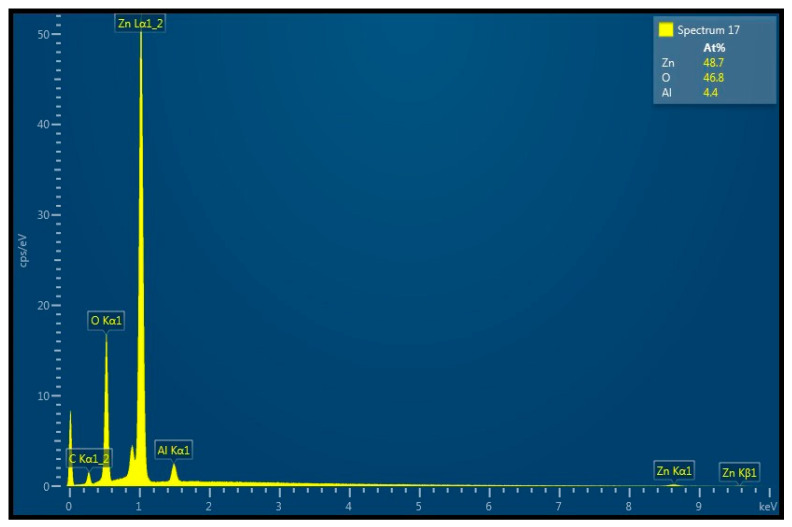
The EDS diagram characterizing a composition of the AZO thin film deposited by the co-sputtering method: the estimated thickness of this film was of ≈860 nm; after deposition, the film was annealed at temperature 500 °C through one hour at for-vacuum conditions (≈5·10^−2^ Torr).

Thin films ZnO:Al were produced by the co-sputtering method using the simultaneous sputtering of two targets, pure ZnO and pure Al, in the argon atmosphere. To prevent the sputtered particles, including oxygen atoms from the ZnO target, from influencing the Al target and oxidizing it, they were separated by a metal screen. The relation between components was controlled by the variation in their sputtering rate. We prepared the films with 2 and 4 atomic percentage of Al in the composition. Figure 4 illustrates the EDS diagram for the ZnO:Al film containing ≈4 atomic percentage of Al. We applied RF power of 200 W for sputtering the ZnO target and DC power of 15 W for the Al target. The estimated thickness of the film was of 860 nm. After deposition, the film was annealed in vacuum conditions (≈5·10^−2^ Torr) through one hour at temperature of 500 °C. 

**Figure 5:**
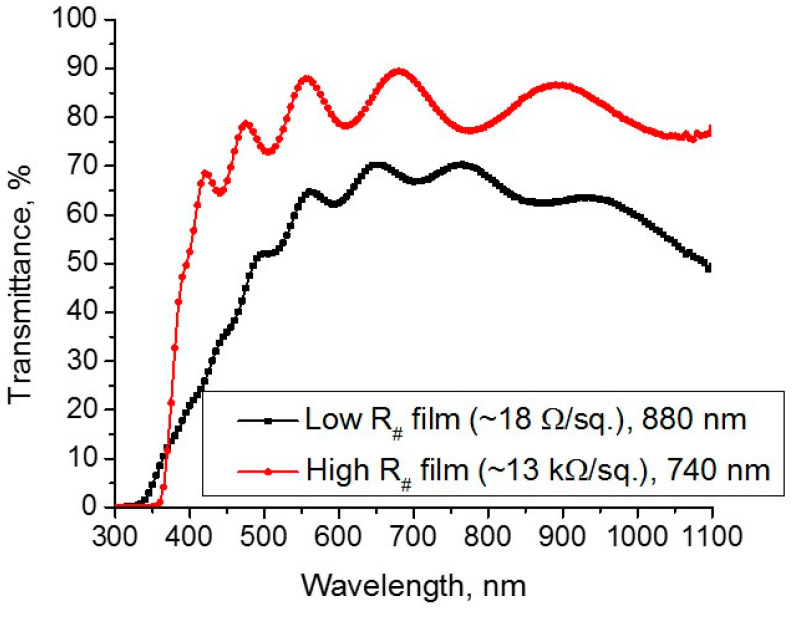
Transmittance characteristics of AZO thin films with low and high resistivity.

Figure 5 represents transmittance characteristics of two films with different resistivity, low and high.

The difference between two curves was the result of different methods of after-deposition thermal treatment. The high-resistivity film was annealed at air atmosphere pumped up to 0.05 Torr at 500 °C during 1 h. The low-resistivity film was annealed in vacuum conditions of 2·10^−6^ Torr during the same time and at the same temperature. As shown in Figure 5, the film annealed at high pressure (0.05 Torr) is more transparent than that explained by good oxidation. The difference in the partial pressure of oxygen in both heat treatment procedures was approximately four orders of magnitude. Thus, oxidation takes place entirely at principal higher pressure, which leads to an increase in transmittance and resistivity of the resulting thin films.

**Figure 6:**
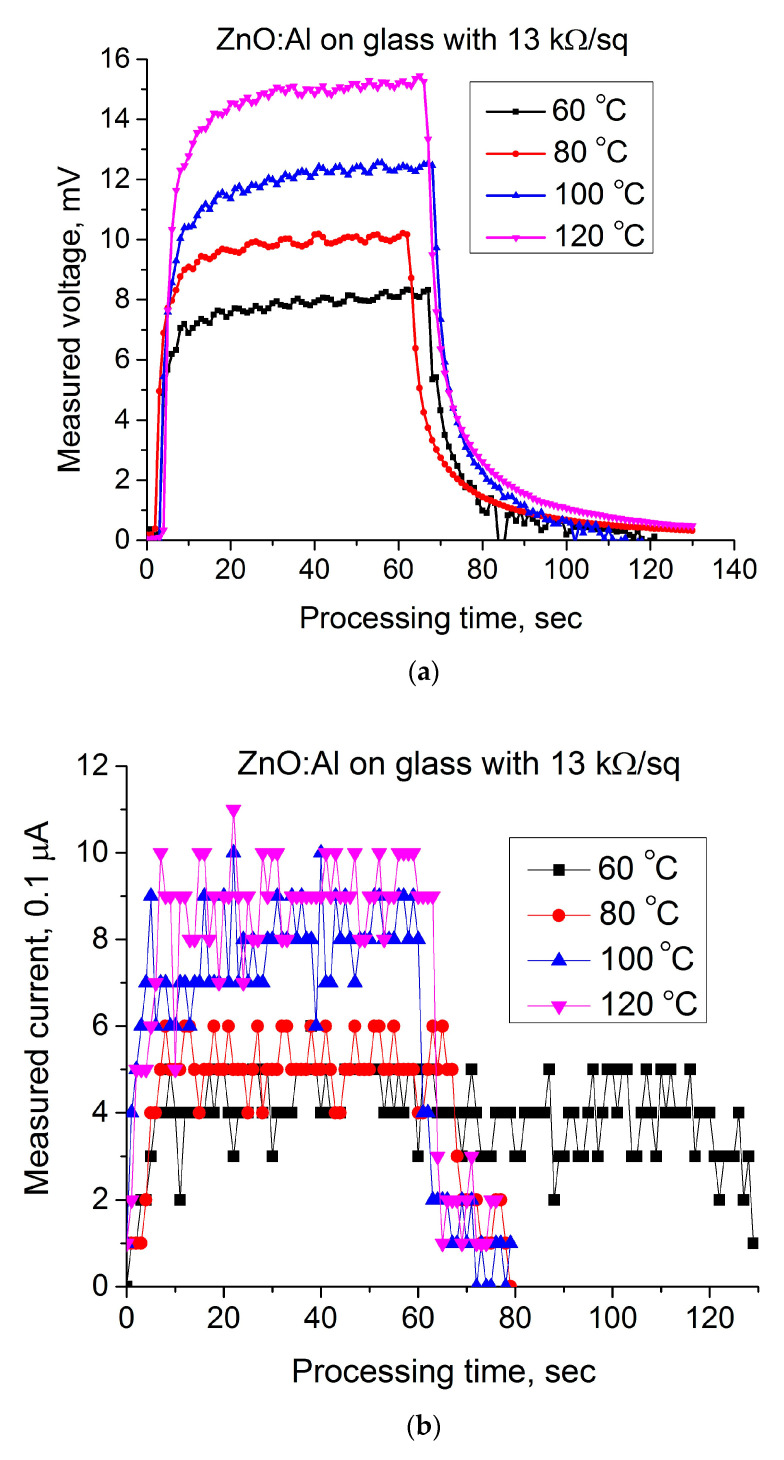
Dynamic hot-probe characteristics of the high-resistive AZO coatings: (**a**) a voltage measured between hot and cold electrodes; (**b**) a current between electrodes.

Figure 6 represents the dynamic hot-probe characteristics measured on the high-resistive ZnO:Al coating deposited on the usual glass slide.

**Figure 7:**
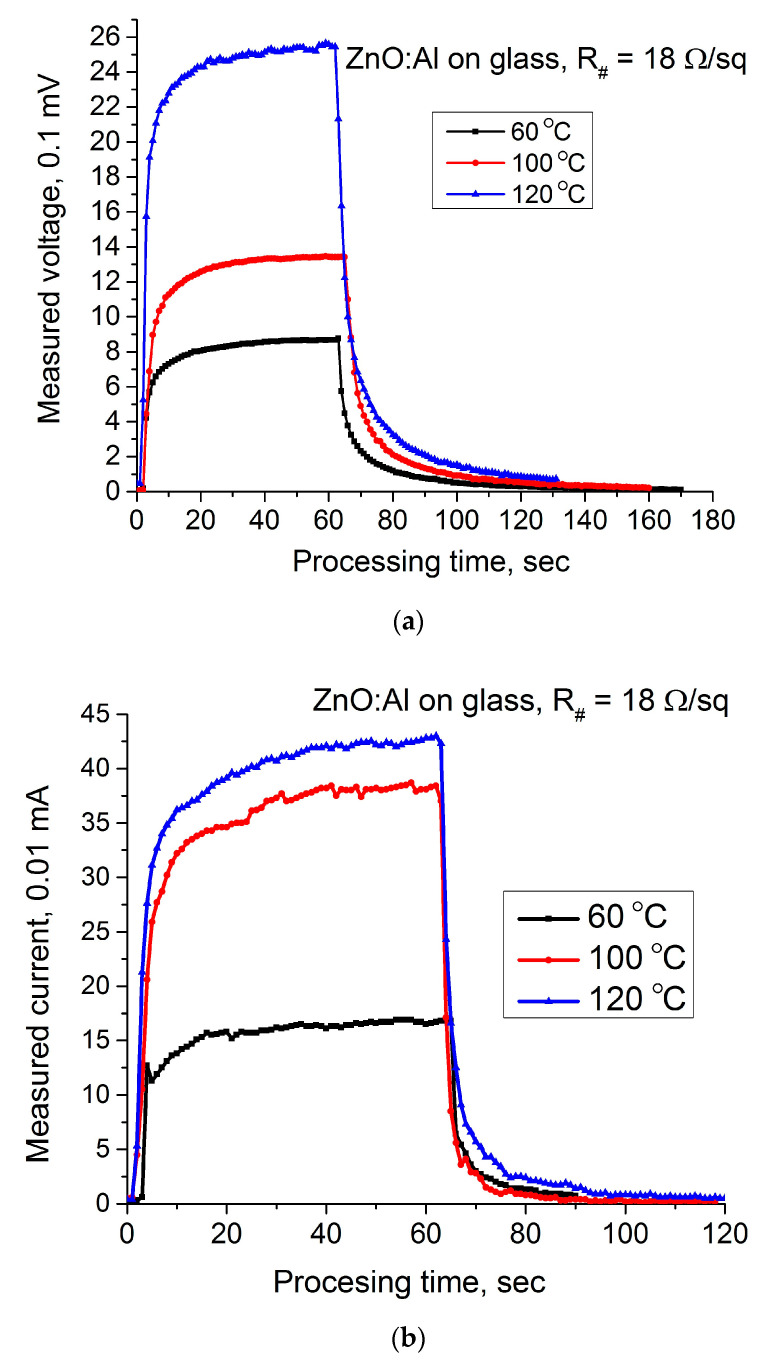
Dynamic hot-probe characteristics of the low-resistive AZO coatings: (**a**) a voltage measured between hot and cold electrodes; (**b**) a current between electrodes.

Figure 6a illustrates the dependence of a hot-electrode voltage on the processing time for different temperatures, and Figure 6b shows the current measured between hot and cold electrodes recorded for the same temperatures. These characteristics are noisy; moreover, the current was measured near the limit of sensitivity of the measuring device. Apparently, the observed oscillations are associated with the high resistivity of measured samples. Obviously, this claim requires statistical proof. This will be the focus of future studies. Figure 7 represents the dynamic hot-probe characteristics measured on the low-resistive ZnO:Al coating deposited on the usual glass slide.

Figure 7a illustrates the dependence of a hot-electrode voltage on the processing time for different temperatures, and Figure 7b presents the current measured between hot and cold electrodes measured for the same temperatures. As it was mentioned above, the difference in these figures is due to the different sheet resistance caused by the different oxidation conditions.

### 3.3. Processing the Experimental Results

Let us consider again Figure 3a recorded for commercial ITO thin films. The measured values of the voltage decreasing after removing a heater from the hot electrode are presented in Table 2. This table also contains a calculation of several parameters, which was performed using the measured data. By definition, the relaxation time may be calculated using a derivative in the first point of the diagram (see Equation (3)):

(25) $$f\left(t\right)=f^{\prime }\left(t_{0}\right)\tau +f\left(t_{i}\right);\;\;\;\tau =-\frac{f\left(t\right)-f\left(t_{i}\right)}{f^{\prime }\left(t_{0}\right)}.$$

Here, $f\left(t_{i}\right)$ is the initial value of the measured parameter, a voltage.

**Figure 8:**
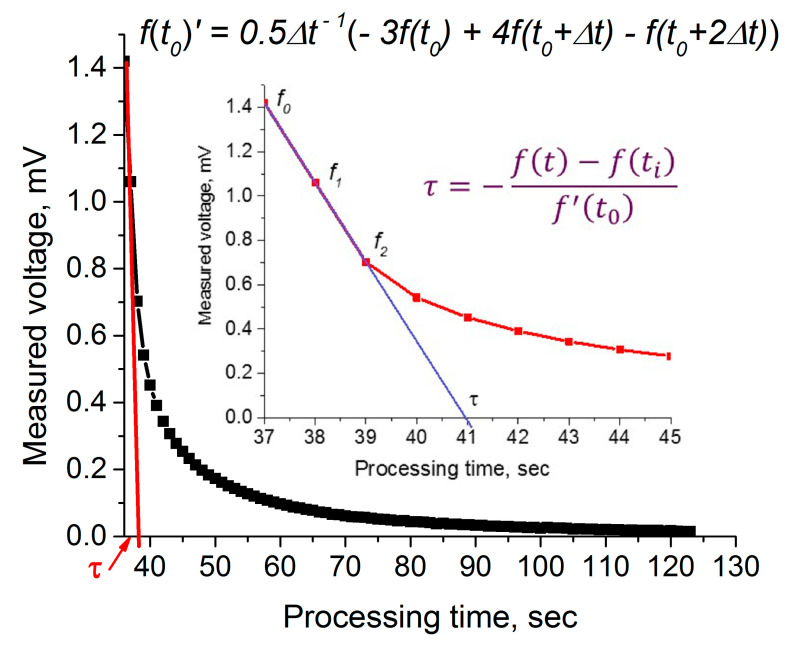
Derivation of the relaxation time from hot-probe measurement; inset shows the characteristic processing.

Now, using the three-point Formula (16) and the Relation (22), we can calculate the relaxation time for all three diagrams, as shown in Figure 8.

**Figure 9:**
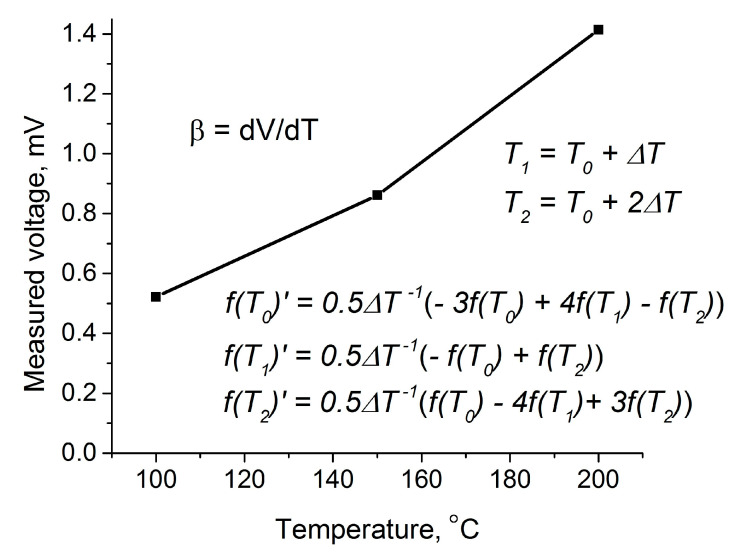
Dependence of the potential difference on the temperature.

The thermopower of the ITO film can be calculated using Figure 9, which shows the dependence of the potential difference on the temperature of the heated electrode.

The results obtained for the mobilities show that they obey Formula (14). So, the main relaxation process in industrial ITO films is lattice scattering or the interaction of excited electrons with phonons.

**Figure 10:**
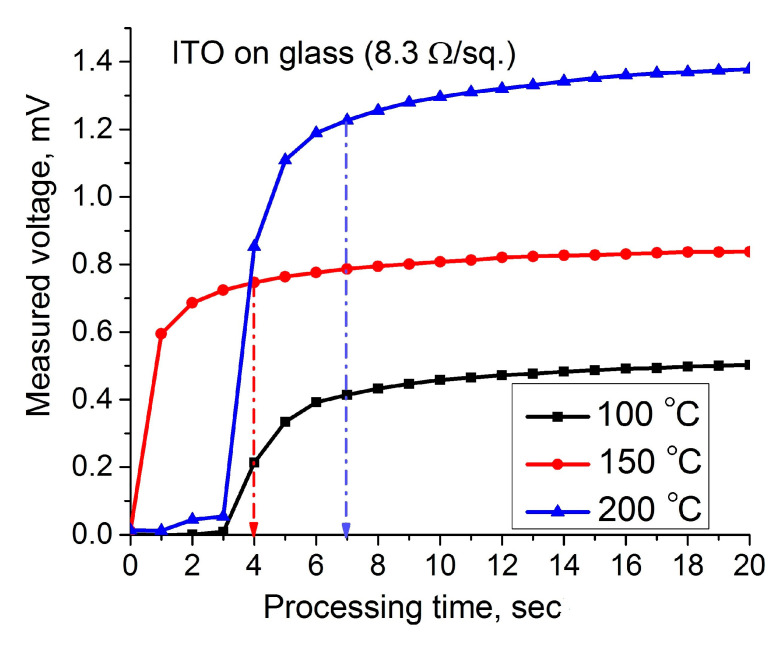
Extraction of saturation parameters, voltage and current, from recorded dynamic hot-probe characteristics.

The calculation of the number of charge carriers moving under thermal excitation can be performed using Formula (21) using the first (ascending) part of the hot-probe characteristic. Figure 10 illustrates the processing of the first part of the recorded hot-probe characteristics.

**Figure 11:**
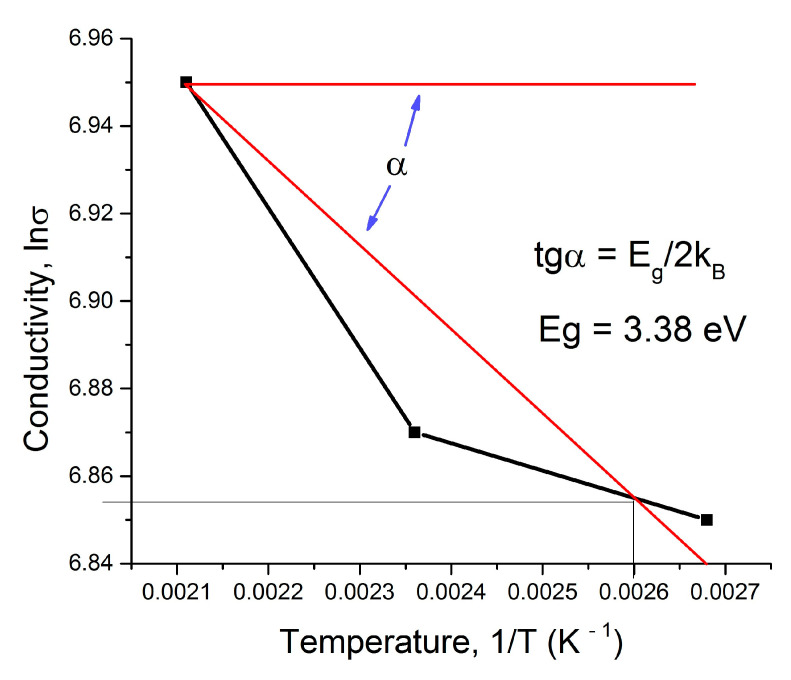
Evaluation of the semiconductor bandgap by experimentally obtained conductivity.

For the calculation, we use the steady-state values of the measured voltage and current reached within a certain short time. These parameters, together with the calculated mobility and thermopower, make it possible to calculate the concentration of charge carriers and the conductivity of the coating by Formula (9). The calculation results are presented in Table 3: 

Comparison of the calculated resistivity of ITO films provided using Formula (9) with the measured value (see Equation (24)) shows good coincidence. Using the calculated conductivity from Table 4 and Table 5 and Arrhenius’s relation [23], we can estimate the bandgap of the ITO layer, as shown in Figure 11:

(26) $$f\left(t\right)=\sigma_{T}=\sigma_{0}e^{\frac{E_{g}}{2k_{B}T}};\;\;\;E_{g}=2k_{B}\frac{ln\sigma_{1}-ln\sigma_{3}}{T_{3}^{-1}-T_{1}^{-1}}.$$

The bandgap of the commercial ITO film calculated by Formula (26) is equal to 3.38 eV.

Now, one can calculate the parameters of AZO experimental samples, which showed two different resistivities: low and high. Table 4, which is similar to Table 2, represents the measured data and calculated parameters for the AZO thin film with high sheet resistance.

The calculation of the concentration of charge carriers and conductivity obtained under thermal activation can be provided by using the table similar to Table 3.

The calculation of parameters of AZO experimental samples with low sheet resistance can be provided by the same scheme. Measured and calculated parameters are given in Table 6.

The calculation of the charge carriers’ concentration and the conductivity obtained under thermal activation is presented in Table 7. 

## 4. Conclusions

The paper presents a method for extracting the main parameters of wide-bandgap TCO thin films from the measured and recorded dynamic hot-probe characteristics and its theoretical foundations. The measurement technique is simple and inexpensive. Conventional measuring instruments equipped with simple software allow the recording and storage of measured data. The technique for extracting the main parameters has been tested on commercial ITO thin films. The results are consistent with the presented and reference data. Then, this method was used to study homemade thin films of AZO, which were prepared by the magnetron co-sputtering method. The parameters of these films such as the majority charge carrier type, concentration, and mobility were extracted from the dynamic hot-probe characteristics measured at different temperatures. The results obtained are in agreement with the literature data for thin AZO films. In addition, the proposed method can be used to calculate the bandgap and thermoelectric power of the films.

## Figures and Tables

**Table 1:** Basic parameters of indium-tin oxide (ITO) and Al-doped ZnO (AZO) thin films.

Parameter	ITO	AZO
Energy gap, Eg (eV)	3.7	3.4
Relative permittivity, $\varepsilon_{r,\infty }$	4	3.66
Static dielectric constant, $\varepsilon_{r,0}$	9	8.91
Electron effective mass, $m_{n}^{*}$ (m_0_)	0.3	0.24
Hole effective mass, $m_{p}^{*}$ (m_0_)	0.22	≈0.7
Typical electron concentration, *n*_c_ (cm^−3^)	≈5·10^20^	≈10^19^
Mobility of electrons, μ_n_ (cm^2^v^−1^s^−1^)	26	50

**Table 2:** Measured voltage and basic calculated parameters of the ITO film.

Parameter	Equation	T_0_ = 373 K (100 °C)	T_1_ = 423 K (150 °C)	T_2_ = 473 K (200 °C)
*V(t*_0_ *)*		0.576	0.865	1.420
*V(t*_1_ *)*		0.368	0.573	1.060
*V(t*_2_ *)*		0.288	0.397	0.703
*y*_0_ *′*	16	−0.272	−0.349	−0.362
τ(s)	25	2.12	2.48	3.92
μ (cm^2^/V·s)	11	14.74	10.90	6.22
β (μV/K)	16	3.12	8.44	13.76

**Table 3:** Measured voltage and basic calculated parameters of the ITO film.

Temperature, *T* (°C)	Rise Time, Δ*t* (s)	Measured Saturation Current, *i_s_* (μA)	Measured Saturation voltage, *U_L_* (mV)	*n*·10^20^ (cm^−3^) Equation (21)	σ (Ω·cm)^−1^ Equation (9)
100	4	11.5	0.414	2.64	0.63·10^3^
150	4	19.6	0.747	3.67	0.64·10^3^
200	4	35.2	1.227	6.93	0.69·10^3^

**Table 4:** Measured voltage and basic calculated parameters of the high-resistance AZO film.

Parameter	Equation	T_0_ = 333 K (60 °C)	T_1_ = 353 K (80 °C)	T_2_ = 373 K (100 °C)
*V(t*_0_ *)*		0.832	1.017	1.246
*V(t*_1_ *)*		0.542	0.872	0.973
*V(t*_2_ *)*		0.432	0.638	0.735
*y*_0_ *′*	16	−0.38	−0.1	−0.29
τ(s)	25	2.19	10.2	4.3
μ (cm^2^/V·s)	11	15.75	3.16	7.27
β (μV/K)	16	8.15	10.4	12.6

**Table 5:** Measured voltage and basic calculated parameters of the high-resistance AZO film.

Temperature, *T* (°C)	Rise Time, Δ*t* (s)	Measured Saturation Current, *i_s_* (μA)	Measured Saturation Voltage, *U_L_* (mV)	*n*·10^18^ (cm^−3^) Equation (21)	σ (Ω·cm)^−1^ Equation (9)
60	6	0.4	0.609	0.76	1.92
80	6	0.5	0.838	3.38	1.71
100	6	0.7	1.037	1.65	1.92

**Table 6:** Measured voltage and basic calculated parameters of the low-resistance AZO film.

Parameter	Equation	T_0_ = 333 K (60 °C)	T_1_ = 373 K (100 °C)	T_2_ = 413 K (140 °C)
*V*(*t*_0_)		0.876	1.341	2.544
*V*(*t*_1_)		0.575	1.099	2.132
*V*(*t*_2_)		0.447	0.882	1.634
*y_0_′*	A18	−0.388	−0.255	−0.369
τ(s)	A25	2.26	5.26	6.89
μ (cm^2^/V·s)	A13	15.26	5.94	4.03
β (μV/K)	A18	3.2	27.8	52.4

**Table 7:** Measured voltage and basic calculated parameters of the low-resistance AZO film.

Temperature, *T* (°C)	Rise Time, Δ*t* (s)	Measured Saturation Current, *i_s_* (μA)	Measured Saturation Voltage, *U_L_* (mV)	*n*·10^18^ (cm^−3^) Equation (21)	σ (Ω·cm)^−1^ Equation (9)
60	6	13.1	0.681	0.17	41.5
100	6	17.1	1.055	0.15	14.3
140	6	28.7	2.133	1.43	92.2

## Data Availability

Not applicable.
